# Epidemiology of spider mite sensitivity: a meta-analysis and systematic review

**DOI:** 10.1186/s13601-018-0209-8

**Published:** 2018-06-18

**Authors:** Ying Zhou, Haoyuan Jia, Xuming Zhou, Yubao Cui, Jun Qian

**Affiliations:** 1 Department of Pediatrics Laboratory, Wuxi Children’s Hospital, Wuxi, 214023 People’s Republic of China; 2 Department of Clinical Laboratory, Wuxi People’s Hospital Affiliated to Nanjing Medical University, No. 299 at Qingyang Road, Wuxi, 214023 Jiangsu Province People’s Republic of China; 3 Department of Pediatrics, Wuxi Children’s Hospital, Wuxi, 214023 People’s Republic of China

**Keywords:** Allergy, Spider mites, *Tetranychus urticae*, *Panonychus citri*, *Panonychus ulmi*

## Abstract

**Background:**

Spider mites, including *Tetranychus urticae*, *Panonychus citri*, and *Panonychus ulmi*, are common pests in gardens, greenhouses, and orchards. Exposure, particularly occupational exposure, to these organisms may lead to the development of respiratory or contact allergies. However, the prevalence of sensitivity to spider mites is unclear.

**Methods:**

We examined the literature to generate an estimate of the global prevalence of allergies to spider mites.

**Results:**

Electronic databases were searched and twenty-three studies reporting the prevalence of sensitivity to spider mites (based on skin prick tests or IgE-based detection systems) in an aggregate total of 40,908 subjects were selected for analysis. The estimated overall rate of spider mite sensitivity was 22.9% (95% CI 19–26.8%). Heterogeneity was high and meta-regression analysis considering variables such as published year, country, number of study subjects, methods for allergen detection (skin prick test, ImmunoCAP, RAST testing, or intradermal test), and mite species revealed no single significant source. Twelve of the 23 studies reported rates of monosensitization (i.e., patients responsive to spider mites but no other tested allergen), yielding a global average of 7% (95% CI 5–9%), hence spider mites represent a unique source of allergens.

**Conclusions:**

Spider mites are an important cause of allergic symptoms. However, the publication bias and heterogeneity evident in this study indicate that further trials using standardized detection methods are needed to determine the association of exposure and symptoms as well as the specific patient characteristics that influence developing spider mite sensitivity.

## Background

The allergenic role of mites of the genus *Dermatophagoides* in indoor floor and mattress dust was discovered in 1967 [1, 2]. Since then, numerous species have been described as the source of allergens capable of sensitizing and inducing allergic symptoms in susceptible and genetically predisposed individuals [3]. The major mites in indoor house dust, *D. pteronyssinus*, *D. farinae*, *Blomia tropicalis*, and *Euroglyphus maynei*, account for 80% of the total allergenic species, with storage mites making up the remainder [4, 5]. Domestic mites, including all indoor mites, belong to the subphylum Chelirata, class Arachnida, subclass Acari, superorder Acariformes, and order Astigmata [6].

Spider mites, also called webspinning mites [7], are common pests in landscapes and gardens and feed on many fruit trees, vines, berries, vegetables, and ornamental plants. All spider mites, belonging to the suborder Prostigmata of the subclass Acari, are outdoor phytophagous mites which cause significant damage to fruit trees throughout the world, causing a considerable economic burden on agriculture [8]. In a Korean study of 2412 patients, 9.8% were sensitized to spider mites [8]. An online search revealed that spider mites are important outdoor allergens that may contribute to work-related asthma and rhinitis in fruit farmers and children living in rural areas and produce a set of allergens that differ from those generated by indoor mites [9]. The aim of our present study was to analyze existing information on the prevalence of spider mite sensitization.

### Search strategy

We have used a search and analysis strategy based on the PRISMA system [10]. To identify related studies published through June 1st, 2017, we performed systematic literature searches of electronic databases including PubMed, the Cochrane Library, EMBASE, Medion, and Web of Science. Search terms were applied by various combinations of Medical Subject Headings (MeSH) and non-MeSH terms as follows: [(spider mite or *Tetranychus* or *Panonychus*) AND (sensitization or allergy or hypersensitivity or specific IgE positive or skin test positive or RAST positive)]. Titles and abstracts identified by electronic searches were examined independently and on screen by two researchers to select potentially relevant studies. Eligibility criteria are given below. Differences were resolved by consensus. A full text paper was obtained wherever possible.

### Eligibility criteria

Studies that investigated the prevalence of sensitivity to spider mites (family Tetranychidae) in full journal articles were selected for review, including cross-sectional, cohort studies, controlled clinical trials and other types. Studies published in conference proceedings, books, book chapters, or research not published in English were excluded.

Eligible studies focused on individuals with allergic disorders defined by in vivo or in vitro tests with mite extract made from *Tetranychus* or *Panonychus* mites. Thus, inclusion into the meta-analysis was restricted to those studies that reported prevalence data for sensitivity to spider mites.

### Data extraction

The following specific information relating to data collection and results was extracted individually from each identified article and entered into a pre-designed Excel spread sheet: data and geographical location, study design, participant inclusion and exclusion criteria, recruitment procedures, number of investigated subjects, age and gender of investigated subjects, occupations or characteristics of the patients, number sensitized to spider mites, detection methods, and mite species. To ensure accuracy, two researchers extracted the data and then compared the results of their extractions.

### Meta-analysis according to the studied population groups

For meta-analysis, the prevalence rates of spider mite sensitization were pooled using the random effects model [11]. Heterogeneity was calculated via Cochran’s Q and τ^2^ tests, and inconsistency is presented as I^2^, which describes the percentage of variability that is due to heterogeneity rather than chance [11].

### Meta-regression analysis

To identify the sources of heterogeneity among studies, meta-regression analysis was carried out [12]. Possible sources of heterogeneity, including published year, country, number of study subjects, methods for allergen detection (skin prick test, ImmunoCAP, RAST testing, or intradermal test), and mite species (*Tetranychus urticae*, *Panonychus ulmi*, or *Panonychus citri*), were included in the analysis.

### Publication bias and meta-analysis

The possibility of publication bias was assessed by graphical analysis of funnel plots. Deeks’ funnel plot asymmetry analysis was performed to identify publication bias [13]. In Deeks’ funnel plots, each data point represents a study, its effect size or prevalence, and the standard error. The meta-analysis was conducted using the Stata v12 software package (Stata Corporation, College Station, TX, USA) and the graphical representation was conducted using forest plots.

## Results

### Characteristics of included studies

**Fig. 1:**
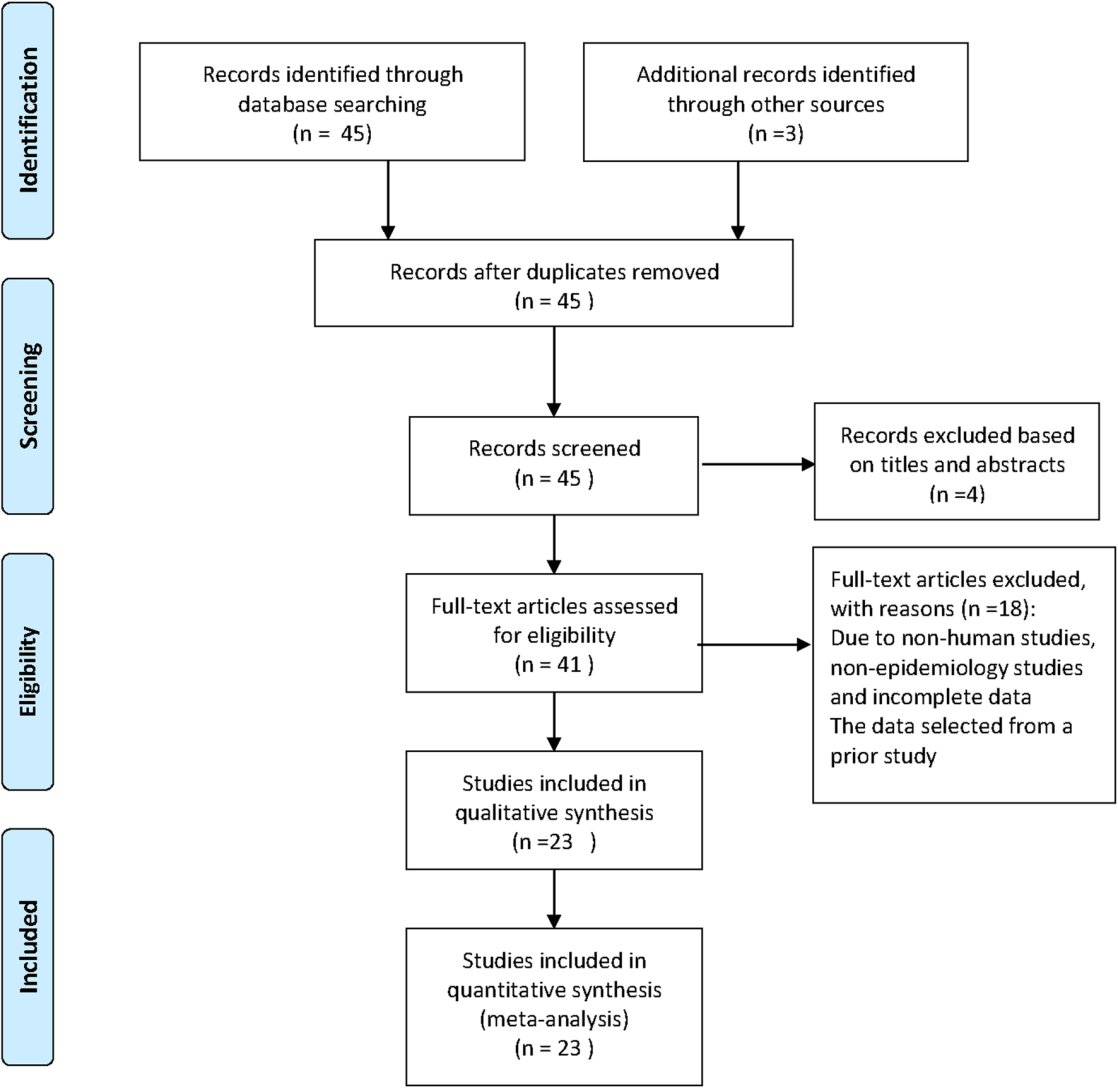
Flow chart of screening and inclusion of studies for review and analysis

Our searches initially retrieved 48 journal article references from electronic databases. Twenty-four of these were subsequently removed due to either duplication or a failure to meet the inclusion criteria. The remaining twenty-four full text articles were then retrieved and critically appraised [8, 9, 14–35]. Of these, the Gargano study [30] was subsequently deleted from the analysis, because this study selected only patients that were SPT+ and tested them to see what percentage had spider mite reactive IgEs. This does not represent an unbiased patient population (since all patients were known to be SPT+). The remaining 23 studies were found to be eligible and were entered into our review and meta-analysis (Fig. 1). Among the 23 included papers, 13 were conducted in Korea, three were conducted in Italy, one was conducted in Japan, two were conducted in Spain, two were conducted in South Africa, and two were conducted in Sweden (Table 1). The sample sizes of the studies entered into the review varied widely from 10 [33] to 8595 [22] with the median sample size being 308. In total, the 23 studies examined 40,908 subjects. Among these 23 papers, Kim et al. [22] reported the prevalence for sensitivity to both *T. urticae* and *P. citri* using separate patient populations. Kim and Lee et al. [25] reported the sensitivity prevalence for both *T. urticae* and *P. ulmi* in the same patient population. For the purposes of meta-analysis, the different mite species were considered separately. Hence, these two studies contributed to two data points.

**Table 1:** The prevalence of spider mite allergy from included studies

Study	Prevalence (%) [95% CI]			% Weight	Country	Sample Size
*Tetranychus urticae*						
Astarita et al. [33]	40.0	9.6	70.4	1.21	Italy	10^f,a^
Astarita et al. [34]	78.3	66.3	90.2	3.30	Italy	46^f,a^
Astarita et al. [35]	6.0	4.5	7.5	4.77	Italy	960^f,a^
Delgado et al. [31]	66.7	47.8	85.5	2.24	Spain	24^f,a^
Jee et al. [29]	32.0	19.1	44.9	3.12	Korea	50^npo^
Jeebhay et al. [28]	22.1	16.2	28.0	4.32	South Africa	190^f,a^
Johansson et al. [27]	25.8	10.4	41.2	2.72	Sweden	31^f,a^
Kim et al. [8]	9.9	8.7	11.1	4.78	Korea	2467^r,u,a^
Kim et al. [22]	4.3	3.9	4.8	4.80	Korea	8595^nao,u,c^
Kim et al. [25]	16.6	13.2	19.9	4.63	Korea	465^f,nao,a^
Kim et al. [24]	19.8	18.0	21.7	4.75	Korea	1806^u^
Kronqvist et al. [21]	24.0	15.4	32.5	3.89	Sweden	96^f,a^
Lee et al. [20]	28.0	26.9	29.0	4.78	Korea	7182^c,a^
Navarro et al. [16]	25.3	19.8	30.8	4.38	Spain	241^f,a^
Seedat et al. [15]	46.0	32.2	59.8	2.98	South Africa	50^u,r,c,a^
Sub-total						
D + L pooled prevalence	27.0	20.5	33.5	56.67		
I–V pooled prevalence	8.7	8.4	9.1			
*Panonychus citri*						
Ashida et al. [32]	83.3	62.2	104.4	1.98	Japan	12^f,a^
Kim et al. [26]	21.8	19.8	23.8	4.74	Korea	1629^nco,c^
Kim et al. [22]	15.6	14.8	16.4	4.79	Korea	8029^nco,c^
Kim et al. [23]	14.3	13.5	15.2	4.79	Korea	6332^r,c^
Kim et al. [14]	23.0	14.8	31.2	3.94	Korea	100^nco,c^
Kim et al. [9]	16.6	11.2	22.0	4.39	Korea	181^f,a^
Lee et al. [19]	14.2	12.1	16.3	4.73	Korea	1037^nco,c^
Lee et al. [18]	1.3	0.60	2.00	4.79	Korea	1000^u,nco,c^
Min et al. [17]	14.9	11.3	18.5	4.61	Korea	375^nco,c^
Sub-total						
D + L pooled prevalence	18.2	12.4	24.0	38.76		
I–V pooled prevalence	10.3	9.9	10.8			
*Panonychus ulmi*						
Kim et al. [25]	23.2	19.4	27.1	4.58	Korea	465^f,naf,a^
Sub-total						
D + L pooled prevalence	23.2	19.4	27.1	4.58		
I–V pooled prevalence	23.2	19.4	27.1			
*Overall*						
D + L pooled prevalence	22.9	19.0	26.8	100.00		
I–V pooled prevalence	9.5	9.2	9.7			

Populations considered in these studies: f, farmers (either outdoor or greenhouse workers); naf, living near apple farms; nco, living near citrus orchards; npo, living near pear orchards; r, rural (unspecified adjacency to specific crop types); u, urban; c, children; a, adults

### Prevalence of spider mite sensitization

**Fig. 2:**
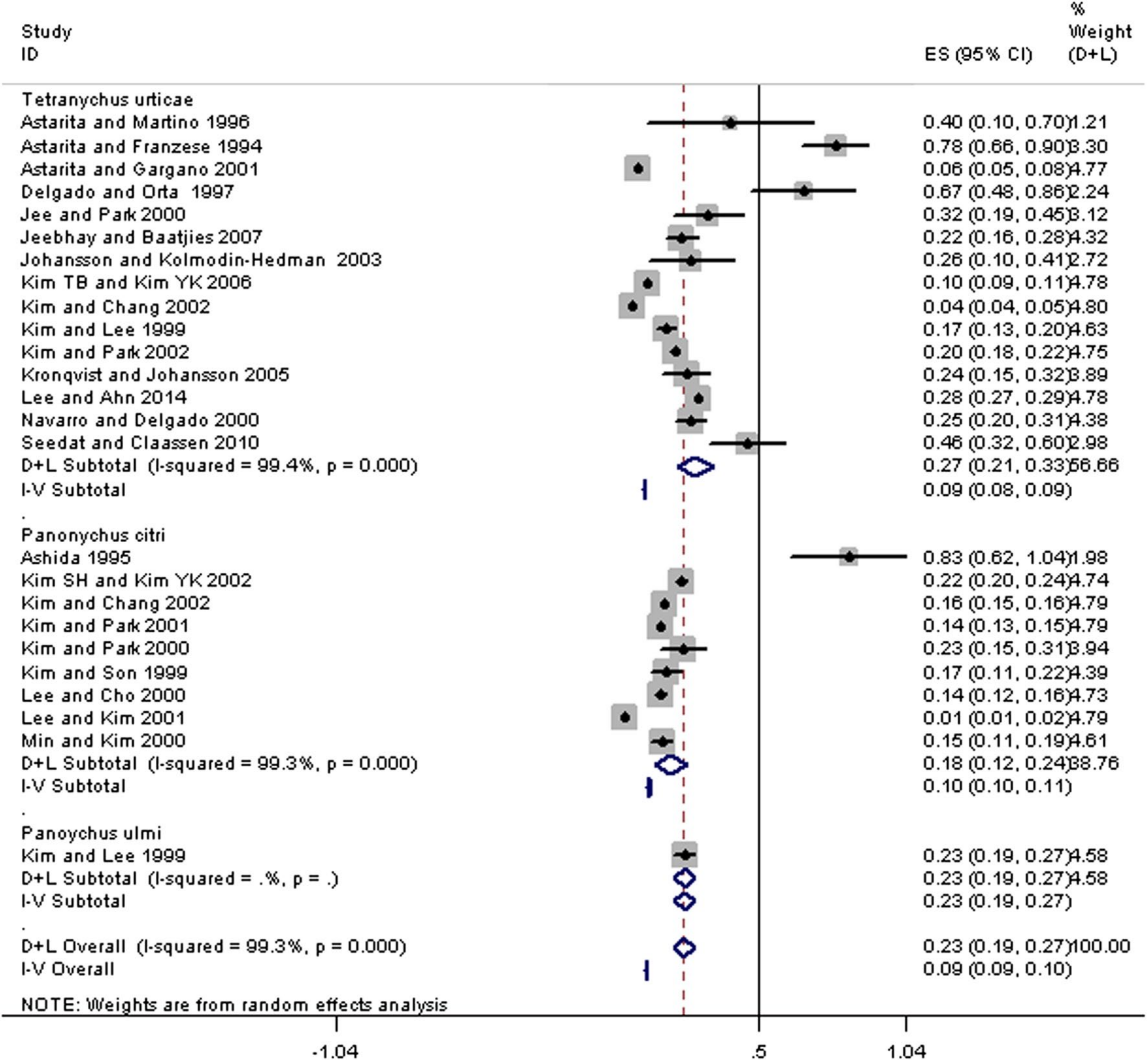
Forest plot of prevalence estimates of spider mite sensitization from included studies

The reported studies included data based on extracts prepared from three spider mite species, i.e., *T. urticae*, *P. citri*, and *P. ulmi* (Table 1). A total of 15 papers reported the prevalence of sensitivity to *T. urticae*, which ranged from 4.3% (95% CI 3.9–4.8%) [22] to 78.3% (95% CI 66.3–90.2%) [34] and reached a global average of 27.0% (95% CI 20.5–33.5%). The heterogeneity found within the studies was high (I^2^ = 99.4%, p < 0.001, Fig. 2 and Table 2). Nine papers reported the prevalence of sensitivity to *P. citri,* which ranged from 1.3% (95% CI 0.6–2%) [18] to 83.3% (95% CI 62.2–104.4%) [32], reaching a global average of 18.2% (95% CI 12.4–24.0%), and the heterogeneity found within the studies was high (I^2^ = 99.3%, p < 0.001, Fig. 2 and Table 2). Only one paper reported the prevalence of sensitivity to *P. ulmi,* which was 23.2% (95% CI 19.4–27.1%). The pooled prevalence estimates of spider mite sensitization to any species was 22.9% (95% CI 19–26.8%).

**Table 2:** Heterogeneity analysis of the involved studies

	Heterogeneity statistic	Degrees of freedom	p	I-squared** (%)	Tau-squared
*Tetranychus urticae*	2177.04	14	< 0.001	99.4	0.0137
*Panonychus citri*	1092.73	8	< 0.001	99.3	0.0070
*Panonychus ulmi*	0.00	0			0.0000
Overall	3351.78	24	< 0.001	99.3	0.0081

Significance test(s) of prevalence = 0

*Tetranychus urticae* z = 8.20, p < 0.001

*Panonychus citri* z = 6.14, p < 0.001

### Publication bias, sensitivity, and meta-regression analysis

**Fig. 3:**
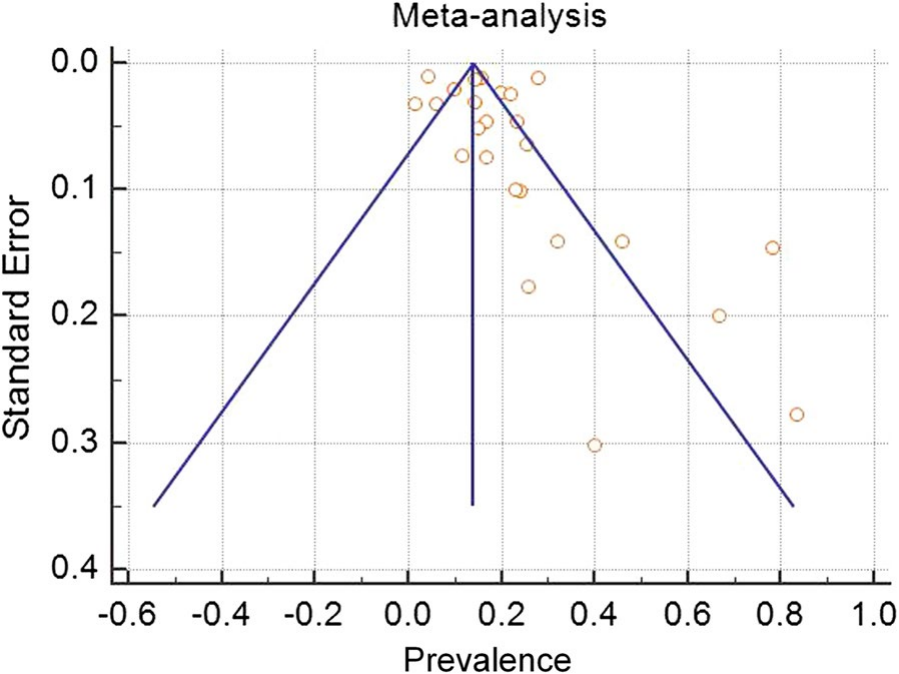
Funnel plot with pseudo 95% confidence limits

**Fig. 4:**
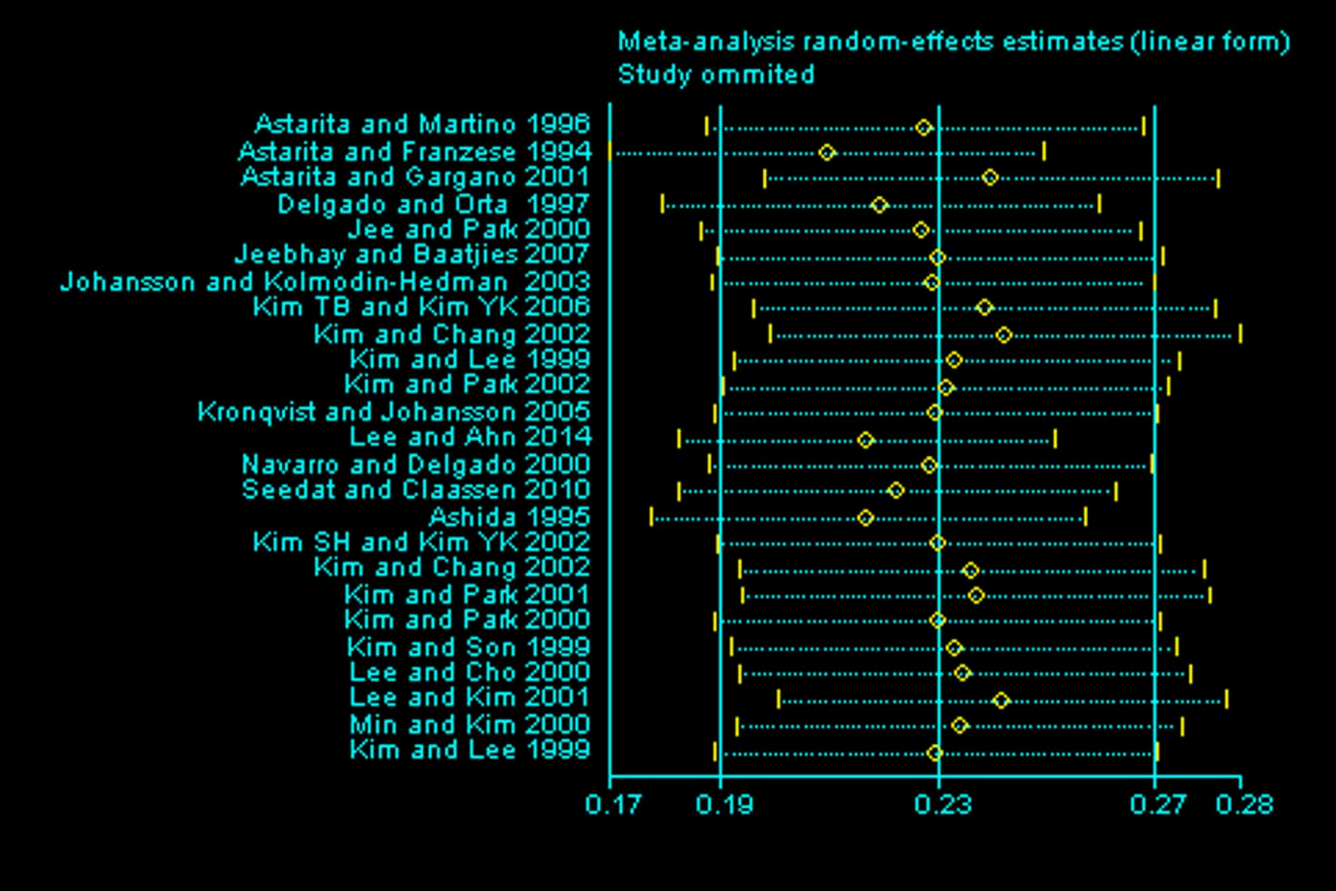
Meta-analysis random-effects estimates for all included studies

Deeks’ funnel plot (Fig. 3) was applied to assess publication bias. In Fig. 3, which shows the prevalence among the cases, the prevalence of the analyzed studies is presented on the x-axis and the standard error of each study is shown on the y-axis. Visual evaluation revealed that the plot was an asymmetric funnel shape, indicating that publication bias was likely present. Figure 4 shows the random effects estimate, with the line representing the calculated median of all samples (0.23) in the middle and lines representing the lower (0.19) and upper (0.27) 95% confidence values to the left and right, respectively. Each circle represents the new mean obtained when the indicated study is removed from the pool. These means all fell within the 95% confidence interval of the total data set, indicating that no individual study had a disproportionate effect on the mean.

As displayed by the forest plot in Fig. 2 and in Table 2, the heterogeneity was significant for *T. urticae* (I^2^ = 99.4%) and *P. citri* (I^2^ = 99.3%). One possible source of heterogeneity was the study population. Eight studies [15, 20, 24, 29, 31–34] enrolled only symptomatic patients (i.e., patients with airway allergy symptoms including asthma and rhinitis or patients with dermatitis) whereas the remaining studies enrolled a mixture of symptomatic and asymptomatic patients. If all symptom-only studies are removed from the sensitivity analysis, the estimated prevalence dropped to 15.43% outside the 95% confidence interval for the total data set (data not shown). From this, we conclude that these studies inflated the mean. However, it is difficult to conclude whether this is due to the patient populations or some other factor. The symptomatic studies typically enrolled fewer patients, so study size might have had an influence. Additionally, when subgroup analysis was performed, the heterogeneity of both the symptomatic and mixed studies was still extremely high (Table 3), indicating that patient populations alone did not contribute much to the overall heterogeneity of the included studies. To examine other sources of heterogeneity, a meta-regression analysis considering the publication year, country, number of study subjects, methods, and mite species analysis was performed, and the results showed that no single analyzed factor could account for the large variability in the reported prevalences. It is likely that a combination of factors makes these studies extremely diverse.

**Table 3:** Effect of population on prevalence of spider mite sensitivity

	N	Number of studies	Prevalence (%)	95% CI	Heterogeneity statistic (Q)	Degrees of freedom	I^2^ (%)
*All studies*							
Overall	40,908	25	23.0	19.0–27.0	2242.56	24	98.8
*Patient population*							
Symptomatic	9180	8	44.0	35.0–53.0	151	7	95.4
Mixed	31,728	17	15.0	12.0–20.0	1354	16	98.82

### Monosensitization to spider mites

**Fig. 5:**
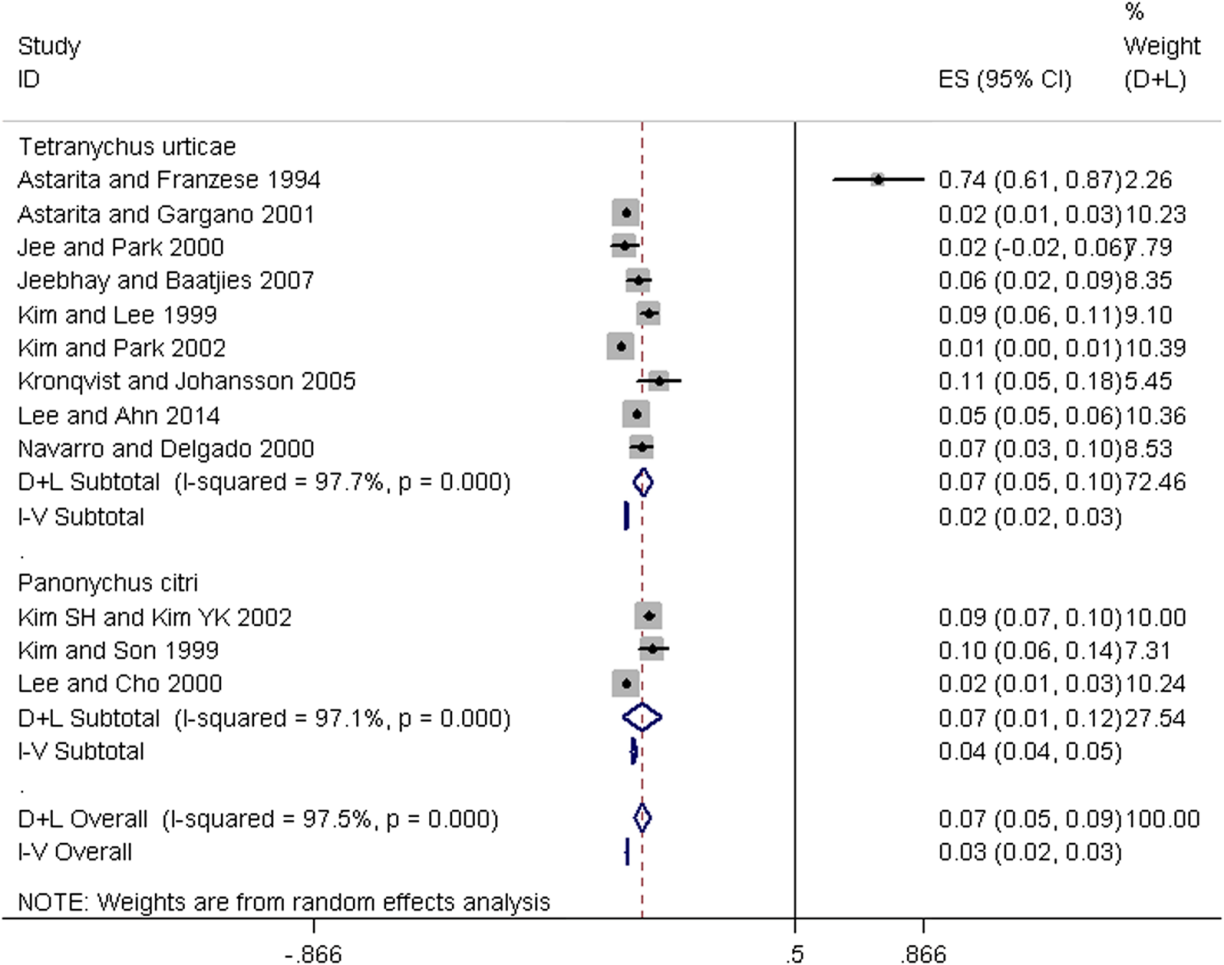
Forest plot of prevalence estimates of monosensitization of spider mite sensitization from included studies

Of the 15 papers reporting the prevalence of sensitivity to *T. urticae* (Table 4), 9 also reported monosensitization rates ranging from 1% (95% CI 0–1%) to 74% (95% CI 61–87%) and reaching a global average of 7% (95% CI 5–10%). The heterogeneity found within the studies was high (I^2^ = 97.7%, p < 0.001). Three papers reported the prevalence of monosensitization to *P. citri,* which was 2% (95% CI 1–3%), 9% (95% CI 7–10%), and 10% (95% CI 6–14%), reaching a global average of 7% (95% CI 1–12.0%), and the heterogeneity within the studies was high (I^2^ = 97.1%, p < 0.001). The pooled prevalence estimate of monosensitization to spider mite sensitization was 7% (95% CI 5–9%) (Fig. 5).

**Table 4:** Studies reporting monosensitization

Study	Monosensitized prevalence (%) [95% CI]			% Weight	Country	Sample size
*Tetranychus urticae*						
Astarita et al. [34]	74.0	61.0	87.0	2.26	Italy	46^f,a^
Astarita et al. [35]	2.1	1.0	3.0	10.23	Italy	960^f,a^
Jee et al. [29]	2.0	− 2.0	6.0	7.79	Korea	50^npo^
Jeebhay et al. [28]	6.0	2.0	9.0	8.35	South Africa	190^f,a^
Kim et al. [25]	8.6	6.0	11.0	9.10	Korea	465^f,nao,a^
Kim et al. [24]	0.7	0.0	1.0	10.39	Korea	1806^u^
Kronqvist et al. [21]	11.0	5.0	18.0	5.45	Sweden	96^f,a^
Lee et al. [20]	5.0	5.0	6.0	10.36	Korea	7182^c,a^
Navarro et al. [16]	7.0	3.0	10.0	8.53	Spain	241^f,a^
Sub-total						
D + L pooled prevalence	7.0	5.0	10.0	72.6		11,036
I–V pooled prevalence	2.0	2.0	3.0			
*Panonychus citri*						
Kim et al. [8]	8.8	7.0	10.0	10.00	Korea	1629^nco,c^
Kim et al. [9]	9.9	6.0	14.0	7.31	Korea	181^f,a^
Lee et al. [19]	2.2	1.0	3.0	10.24	Korea	1037^nco,c^
Sub-total						
D + L pooled prevalence	7.0	1.0	12.0	27.54		2847
I–V pooled prevalence	4.0	4.0	5.0			
*Overall*						
D + L pooled prevalence	7.0	5.0	9.0	100		13,883
I–V pooled prevalence	3.0	2.0	3.0			

Populations considered in these studies: f, farmers (either outdoor or greenhouse workers); naf, living near apple farms; nco, living near citrus orchards; npo, living near pear orchards; r, rural (unspecified adjacency to specific crop types); u, urban; c, children; a, adults

## Discussion

This review provides the first comprehensive search and synthesis of the international literature on the prevalence of spider mite sensitization. The result of our synthesis of all prevalence estimates was 22.9% (95% CI 19.0–26.8%) but may be higher when only symptomatic patients are considered [43.9% (95% CI 35.1–52.9%)]. Our pooled estimate indicates that spider mite sensitivity is moderately common in farming populations. Mite subgroup prevalence estimates were 27% (95% CI 20.5–33.5%) for *T. urticae* sensitivity and 18.2% (95% CI 12.4–24.0%) for *P. citri* sensitivity. Only one paper reported the prevalence of *P. ulmi* sensitivity. Therefore, agricultural workers dealing with fruit trees or working in greenhouses as well as in the surrounding rural population are at risk for developing sensitivity to *T. urticae* and *P. citri*. Further studies are needed to confirm the prevalence of *P. ulmi* sensitivity and to examine if sensitivity to spider mites is a cause of occupational allergies and/or general allergies in rural populations.

The overall sensitivity estimates include patients reactive to spider mite species who may also be sensitized to other environmental allergens. For such polysensitized individuals, a positive skin reaction to spider mites could indicate a primary allergic response or cross-reactivity. To address this, some studies reported the rates of mono-sensitization (defined as reactivity to spider mites but no other tested allergen). Our pooled prevalence estimate for monosensitization to spider mites was 7% (95% CI 5–9.0%), and subgroup prevalence estimates were 7% (95% CI 5–10%) for *T. urticae* sensitivity and 7% (95% CI 1–12.0%) for *P. citri* sensitivity. This indicates that spider mites are the primary sensitizing agent for a moderate number of individuals living primarily in rural settings. Jee et al. used competitive ELISAs and found that *D. pteronyssinus* extracts could not compete with IgE binding to *T. urticae* proteins in serum from a mono-sensitized patient but could compete in serum from polysensitized patients. Unfortunately, little progress has been made in identifying spider mite-specific antigens. Studies have used SDS-PAGE and IgE-immunoblotting to identify 20 [31], 24 [27] and 10 [36] IgE-reactive bands in spider mite extracts, but which of these components are species specific has yet to be determined. Additionally, it should be noted that patients sensitized to other allergens (including domestic mites and/or non-taxonomically related species) are more likely to also be reactive to spider mites [9, 16, 19, 25, 35]. This generalized atopy is known to be true for a variety of high molecular weight allergens and is believed to indicate a hyper-reactive IgE response in certain sensitive patients [37].

The authors believe that the searches conducted were comprehensive and the sensitivity analysis demonstrates that the calculated mean was not unduly influenced by a single study, and thus our findings are generally robust. However, publication bias is present based on the asymmetric funnel plot, and the heterogeneity of the studies was quite large. The heterogeneity observed could come from the different study settings and populations. The estimated prevalence of spider mite sensitization in symptomatic patients was 43.9% (95% CI 35.1–52.9%) which was 2.9 times higher than that found in mixed populations [15.4% (95% CI 11.6–19.7%)]. Heterogeneity was, however, still very high within the subgroups, hence these results should be interpreted cautiously. Some studies reporting data from mixed populations did detect an association between spider mite reactivity and symptoms, but others did not. For example, using patient subgroup information published by Kim and Son et al. [9] revealed that, in this study, the prevalence of spider mite allergies in symptomatic patients was 4 times higher than that in asymptomatic patients. However, in Lee et al. [19], prevalence estimates were similar in symptomatic and non-symptomatic patients. Of note, several studies tested the onset of symptoms in response to a *T. urticae* challenge. Astarita et al. [34] examined the onset of allergic symptoms and tracked the peak expiratory flow rate in spider mite-sensitive patients exposed to an infested green-house environment, and two studies [29, 31] performed a bronchial challenge with *T. urticae* extracts and observed responses in the majority of *T. urticae*-sensitive patients. This indicates that spider mite sensitivity has clinical relevance, but this may vary based on the location and population being considered.

We investigated other possible sources of heterogeneity with meta-regression analyses but could not identify a single factor responsible for the variation. Two factors that may be relevant based on individual studies are patient age and site of residence. Kim et al. [8] reported that the sensitization rate to *T. urticae* increased with age, and Kim et al. [22] reported that the prevalence of spider mite allergies in rural areas was higher than the prevalence in urban settings. In regards to age, few studies of *T. urticae* sensitivity included children (Table 1), whereas the majority of the *P. citri* studies only enrolled children. This could account for the lower prevalence of sensitivity in the *P. citri* studies, or it could indicate that *P. citri* is a weaker sensitizing agent.

## Conclusions

In brief, spider mites are important sensitizing agents particularly in farming populations where contact is the most likely. In some of the reviewed studies, the prevalence of spider mite sensitivity was reported to be higher in patients with allergic symptoms (particularly occupational allergies), and thus exposure may correlate with disease. The moderate prevalence of spider mite monosensitization indicates that these organisms produce unique allergens, and thus specific diagnostic tests and treatment regimens for spider mite sensitization are likely warranted. These conclusions should, however, be interpreted cautiously. Publication bias was present, the heterogeneity of the analyzed studies was extremely high, and the sources contributing to this heterogeneity were unclear. Additional cross-sectional studies using more standardized protocols are needed to assess how specific patient characteristics influence the acquisition of spider mite sensitization and whether and how this progresses to allergic disease.

## References

[1] Voorhorst R, Spieksma FTM, Varekamp H, Leupen MJ, Lyklema AW (1967). The house-dust mite (*Dermatophagoides pteronyssinus*) and the allergens it produces. Identity with the house-dust allergen. J Allergy.

[2] Fernández-Caldas E, Puerta L, Caraballo L (2014). Mites and allergy. Chem Immunol Allergy.

[3] Thomas WR (2016). House dust mite allergens: new discoveries and relevance to the allergic patient. Curr Allergy Asthma Rep.

[4] Caraballo L (2017). Mite allergens. Expert Rev Clin Immunol.

[5] Calderón MA, Kleine-Tebbe J, Linneberg A, De BF, Hernandez FRD, Virchow JC, Demoly P (2015). House dust mite respiratory allergy: an overview of current therapeutic strategies. J Allergy Clin Immunol Pract.

[6] Appel HM, Kunick F, Hoffmann TK, Greve J (2016). Sensitization against domestic mites when perennial nasal symptoms are present. Allergologie.

[7] Manson DCM, Gerson U, Lindquist EE, Sabelis MW, Bruin J (1996). Web spinning, wax secretion and liquid secretion by eriophyoid mites. World crop pests.

[8] Kim TB, Kim YK, Chang YS, Kim SH, Hong SC, Jee YK, Cho SH, Min KU, Kim YY (2006). Association between sensitization to outdoor spider mites and clinical manifestations of asthma and rhinitis in the general population of adults. J Korean Med Sci.

[9] Kim YK, Son JW, Kim HY, Park HS, Lee MH, Cho SH, Min KU, Kim YY (1999). Citrus red mite (*Panonychus citri*) is the most common sensitizing allergen of asthma and rhinitis in citrus farmers. Clin Exp Allergy.

[10] Moher D, Liberati A, Tetzlaff J, Altman DG, PRISMA Group (2009). Preferred reporting items for systematic reviews and meta-analyses: the PRISMA statement. PLoS Med.

[11] Higgins JP, Thompson SG, Deeks JJ, Altman DG (2003). Measuring inconsistency in meta-analyses. BMJ.

[12] Devillé WL, Buntinx F, Bouter LM, Montori VM, de Vet HC, Da VDW, Bezemer PD (2002). Conducting systematic reviews of diagnostic studies: didactic guidelines. BMC Med Res Methodol.

[13] Deeks JJ, Macaskill P, Irwig L (2005). The performance of tests of publication bias and other sample size effects in systematic reviews of diagnostic test accuracy was assessed. J Clin Epidemiol.

[14] Kim YK, Park HW, Park HS, Kim HY, Kim SH, Bai JM, Cho SH, Kim YY, Min KU (2000). Sensitivity to citrus red mite and the development of asthma. Ann Allergy Asthma Immunol.

[15] Seedat RY, Claassen J, Claassen AJ, Joubert G (2010). Mite and cockroach sensitisation in patients with allergic rhinitis in the Free State. S Afr Med J.

[16] Navarro AM, Delgado J, Sanchez MC, Orta JC, Martinez A, Palacios R, Martinez J, Conde J (2000). Prevalence of sensitization to *Tetranychus urticae* in greenhouse workers. Clin Exp Allergy.

[17] Min KU, Kim YK, Park HS, Lee MH, Lee BJ, Son JW, Kim YY, Cho SH (2000). Bronchial responsiveness to methacholine is increased in citrus red mite (*Panonychus citri*)-sensitive children without asthmatic symptoms. Clin Exp Allergy.

[18] Lee MH, Kim YK, Min KU, Lee BJ, Bahn JW, Son JW, Cho SH, Park HS, Koh YY, Kim YY (2001). Differences in sensitization rates to outdoor aeroallergens, especially citrus red mite (*Panonychus citri*), between urban and rural children. Ann Allergy Asthma Immunol.

[19] Lee MH, Cho SH, Park HS, Bahn JW, Lee BJ, Son JW, Kim YK, Koh YY, Min KU, Kim YY (2000). Citrus red mite (*Panonychus citri*) is a common sensitizing allergen among children living around citrus orchards. Ann Allergy Asthma Immunol.

[20] Lee JE, Ahn JC, Han DH, Kim DY, Kim JW, Cho SH, Park HW, Rhee CS (2014). Variability of offending allergens of allergic rhinitis according to age: optimization of skin prick test allergens. Allergy Asthma Immunol Res.

[21] Kronqvist M, Johansson E, Kolmodin-Hedman B, Oman H, Svartengren M, van Hage-Hamsten M (2005). IgE-sensitization to predatory mites and respiratory symptoms in Swedish greenhouse workers. Allergy.

[22] Kim YK, Chang YS, Lee MH, Hong SC, Bae JM, Jee YK, Chun BR, Cho SH, Min KU, Kim YY (2002). Role of environmental exposure to spider mites in the sensitization and the clinical manifestation of asthma and rhinitis in children and adolescents living in rural and urban areas. Clin Exp Allergy.

[23] Kim YK, Park HS, Kim HY, Jee YK, Son JW, Bae JM, Lee MH, Cho SH, Min KU, Kim YY (2001). Citrus red mite (*Panonychus citri*) may be an important allergen in the development of asthma among exposed children. Clin Exp Allergy.

[24] Kim YK, Park HS, Kim HA, Lee MH, Choi JH, Kim SS, Lee SK, Nahm DH, Cho SH, Min KU, Kim YY (2002). Two-spotted spider mite allergy: immunoglobulin E sensitization and characterization of allergenic components. Ann Allergy Asthma Immunol.

[25] Kim YK, Lee MH, Jee YK, Hong SC, Bae JM, Chang YS, Jung JW, Lee BJ, Son JW, Cho SH (1999). Spider mite allergy in apple-cultivating farmers: European red mite (*Panonychus ulmi*) and two-spotted spider mite (*Tetranychus urticae*) may be important allergens in the development of work-related asthma and rhinitis symptoms. J Allergy Clin Immunol.

[26] Kim SH, Kim YK, Lee MH, Hong SC, Bae JM, Min KU, Kim YY, Cho SH (2002). Relationship between sensitization to citrus red mite (*Panonychus citri*) and the prevalence of atopic diseases in adolescents living near citrus orchards. Clin Exp Allergy.

[27] Johansson E, Kolmodin-Hedman B, Kallstrom E, Kaiser L, van Hage-Hamsten M (2003). IgE-mediated sensitization to predatory mites in Swedish greenhouse workers. Allergy.

[28] Jeebhay MF, Baatjies R, Chang YS, Kim YK, Kim YY, Major V, Lopata AL (2007). Risk factors for allergy due to the two-spotted spider mite (*Tetranychus urticae*) among table grape farm workers. Int Arch Allergy Immunol.

[29] Jee YK, Park HS, Kim HY, Park JS, Lee KY, Kim KY, Kim YK, Cho SH, Min KU, Kim YY (2000). Two-spotted spider mite (*Tetranychus urticae*): an important allergen in asthmatic non-farmers symtomatic in summer and fall months. Ann Allergy Asthma Immunol.

[30] Gargano D, Romano C, Manguso F, Cutajar M, Altucci P, Astarita C (2002). Relationship between total and allergen-specific IgE serum levels and presence of symptoms in farm workers sensitized to *Tetranychus urticae*. Allergy.

[31] Delgado J, Orta JC, Navarro AM, Conde J, Martinez A, Martinez J, Palacios R (1997). Occupational allergy in greenhouse workers: sensitization to *Tetranychus urticae*. Clin Exp Allergy.

[32] Ashida T, Ide T, Tabata S, Kunimatsu M, Etoh Y, Yoshikawa T, Matsunaga T (1995). IgE-mediated allergy to spider mite, *Panonychus citri* in occupationally exposed individuals. Arerugi.

[33] Astarita C, Di Martino P, Scala G, Franzese A, Sproviero S (1996). Contact allergy: another occupational risk to *Tetranychus urticae*. J Allergy Clin Immunol.

[34] Astarita C, Franzese A, Scala G, Sproviero S, Raucci G (1994). Farm workers’ occupational allergy to *Tetranychus urticae*: clinical and immunologic aspects. Allergy.

[35] Astarita C, Gargano D, Manguso F, Romano C, Montanaro D, Pezzuto F, Bonini S, Altucci P, Abbate G (2001). Epidemiology of allergic occupational diseases induced by *Tetranychus urticae* in greenhouse and open-field farmers living in a temperate climate area. Allergy.

[36] Kim YK, Oh SY, Jung JW, Min KU, Kim YY, Cho SH (2001). IgE binding components in *Tetranychus urticae* and *Panonychus ulmi*-derived crude extracts and their cross-reactivity with domestic mites. Clin Exp Allergy.

[37] Osterman K, Zetterstrom O, Johansson SG (1982). Coffee worker’s allergy. Allergy.

